# Orangutan information broadcast via consonant-like and vowel-like calls breaches mathematical models of linguistic evolution

**DOI:** 10.1098/rsbl.2021.0302

**Published:** 2021-09-29

**Authors:** Adriano R. Lameira, António Alexandre, Marco Gamba, Matthew G. Nowak, Raquel Vicente, Serge Wich

**Affiliations:** ^1^ Department of Psychology, University of Warwick, Coventry, UK; ^2^ School of Psychology and Neuroscience, University of St Andrews, Scotland, UK; ^3^ Independent researcher, University of Turin, Turin, Italy; ^4^ Department of Life Sciences and Systems Biology, University of Turin, Turin, Italy; ^5^ Sumatran Orangutan Research Programme, PanEco-YEL, North Sumatra, Indonesia; ^6^ Department of Anthropology, Southern Illinois University, Carbondale, IL, USA; ^7^ School of Natural Sciences and Psychology, Liverpool John Moores University, Liverpool, UK; ^8^ Institute for Biodiversity and Ecosystem Dynamics, University of Amsterdam, Amsterdam, The Netherlands

**Keywords:** language origin, language evolution, proto-consonants, proto-vowels, great apes, orangutans (*Pongo* spp.)

## Abstract

The origin of language is one of the most significant evolutionary milestones of life on Earth, but one of the most persevering scientific unknowns. Two decades ago, game theorists and mathematicians predicted that the first words and grammar emerged as a response to transmission errors and information loss in language's precursor system, however, empirical proof is lacking. Here, we assessed information loss in proto-consonants and proto-vowels in human pre-linguistic ancestors as proxied by orangutan consonant-like and vowel-like calls that compose syllable-like combinations. We played back and re-recorded calls at increasing distances across a structurally complex habitat (i.e. adverse to sound transmission). Consonant-like and vowel-like calls degraded acoustically over distance, but no information loss was detected regarding three distinct classes of information (*viz.* individual ID, context and population ID). Our results refute prevailing mathematical predictions and herald a turning point in language evolution theory and heuristics. Namely, explaining how the vocal–verbal continuum was crossed in the hominid family will benefit from future mathematical and computational models that, in order to enjoy empirical validity and superior explanatory power, will be informed by great ape behaviour and repertoire.

## 1. Introduction

Communication in natural (e.g. human language) and artificial systems (e.g. computer language) rests on three vertices: the encoder, the decoder and the communication channel linking the two [1]. With regards to language origin—the last major evolutionary transition of life on Earth [2]—much attention has been dedicated to the role of the encoder (its anatomical [3–6] and motoric attributes [7–11]), the receiver (its anatomical [12,13] and perceptual attributes [14–18]) and the interactions between the two [19]. Surprisingly, however, the role of the channel [1]—the interval between encoder and decoder that a signal must traverse—in the emergence of language has remained virtually ignored [20].

This knowledge gap is particularly problematic in light of game theory and mathematical models of language evolution [21–23]. Notably, these models have predicted that the first words and grammatical rules emerged to minimize error and information loss in language's precursor channel. Regarding word origin, this argument asserts that the lengthier a signal combination, the lower the probability of mistaking signals for each other. Regarding syntax origin, it asserts that the more varied a sequence of signal combinations, the lower the probability of mistaking the events being referred to, with words and syntax having, thus, developed in the human lineage to decrease transmission errors. Without basic knowledge about the communication channel used by our ancestors to broadcast information and its ‘error limit’ [21–23], it is impossible, however, to validate these models or their proposed evolutionary scenario.

Human evolution unfolded in parallel with acute climate and ecological changes in the African continent [24], however, it is unclear when and where the first forms of language manifested among human ancestors. Regardless of whether proto-language originated in the rainforest, woodland or savannah, the hypothesis that the first linguistic structures emerged to avert error can be best tested in forested habitats, which pose the most adverse conditions to sound transmission, and thus, where signal and information limits can be assessed.

To implement an empirical proof of the currently prevailing mathematical models of linguistic evolution, we assessed information loss in wild orangutan voiceless consonant-like and voiced vowel-like calls [7]. These calls exhibit articulatory homology with their human counterparts, and therefore, represent living proxies of spoken language's putative pre-linguistic units [25–27]. Namely, we played back consonant-like ‘kiss-squeaks’ and vowel-like ‘grumphs’ [28] and re-recorded these calls at increasing distances. Critically, bar humans, orangutans are the only known great ape to produce consonant-like and vowel-like calls combined into syllable-like combinations [29], therefore, presenting a privileged hominid model for this study [30].

## 2. Material and methods

### (a) In brief

Calls were originally recorded from wild orangutan individuals across contexts and populations of Sumatran (*Pongo abelii*) and Bornean orangutans (*Pongo pygmaeus*). Only consonant- and vowel-like calls that were from the same syllable-like combination were used for playback. We extracted four acoustic parameters over distance. We used individual, contextual and geographical acoustic signatures [25] to assess information loss. This set-up mimicked the putative proto-combinatoric conditions at the moment of language origin. Methodologically, this allowed us to control for biasing factors between consonant- and vowel-like calls (e.g. individuals, context, recording settings).

### (b) Study site

Playback experiments were conducted at the Sikundur Research Station (3°55′48.07″N; 98°2′31.17″E), Leuser Ecosystem, North Sumatra, Indonesia. The Sikundur forest is located on the eastern forest margin of the Alas River dividing the Leuser Ecosystem along its north–south axis and constituting a major dispersal barrier for orangutans at this altitude [31]. Presently, the forest is a dipterocarp tropical rainforest, comprising disturbed primary forest and secondary/regrowth forest, which was the target of previous logging operations (between 1970 and 1980, and later during the 1990s [32]).

### (c) Data collection

Recordings for the playback playlist were previously collected at three research stations: Tuanan and Gunung Palung (Central and West Kalimantan, respectively, Indonesian Borneo) and Sampan Getek (North Sumatra, Indonesia). The playback playlist included 120, 118 and 249 calls to assess individual ID, context and population ID information, respectively (see more in electronic supplementary material). Orangutan kiss-squeaks [28] were used as living proxies of voiceless proto-consonants, orangutan grumphs [28] as living proxies of voiced proto-vowels.

All kiss-squeaks and grumphs were selected from call combinations composed of the two calls, specifically kiss-squeak + grumph (see 'Data analyses' (§2e) and electronic supplementary material). All recordings were set to the same peak amplitude prior to playback using Raven interactive sound analysis (v. 1.2.1, Cornell Lab of Ornithology, Ithaca, New York). No further signal transformations were conducted.

Playbacks were conducted using a Marantz Digital Recorder PMD-660 (D&M Holdings, Kawasaki, Japan) connected to a Nagra DSM speaker (Audio Technology Switzerland S.A., Romanel, Switzerland). The speaker was set at 1–1.5 m from the ground. Because Sikundur is partially a regrowth/secondary forest, with abundant undergrowth below the understorey, this height offered a suitable means to explore the effects of complex habitat structure on broadcast performance. Playback volume was set at approximately 100 dB SPL at 1 m distance to facilitate assessment of sound degradation over distance and was not meant to emulate orangutan natural vocal loudness. Playbacks were conducted between 5.30 and 6.30 local time in the absence of wind and with no rain during the previous 48 h. This time was elected for playbacks because, in this habitat, early mornings were the time of day with the least biotic noise. We made no presumptions as to whether early human ancestors communicated predominantly at this time. All recordings along the same transect were conducted in the same morning.

Playbacks were conducted twice, at two locations (i.e. along two transects), i.e. once at each location. Re-recordings were conducted every 25 m along the two transects across the forest up until 100 m away, at which point playbacks became too faint to be analysed. Transects started within 10 m from each other and advanced forward in an oblique direction one from other. Using different transects allowed us to assess the impact of particular phonological features (e.g. larger tree trucks, leaf density) on broadcast performance. Transects were straight, flat and included no obvious canopy openings or clearings. Playbacks were re-recorded using a ZOOM H4next Handy Recorder (ZOOM Corporation, Tokyo, Japan) connected to a RØDE NTG-2 directional microphone (RØDE LLC, Sydney, Australia). Audio data were recorded using the WAVE PCM format at 16 bits. The microphone was set at 1–1.5 m from the ground. Data for distance zero were extracted from the original playback recordings. In total, 7826 calls (incl. original at 0 m and re-recordings up to 100 m) were collected (see electronic supplementary material, for sample breakdown). For each transect, three playbacks sessions were conducted, one for each information type: one playlist comprised recordings varying in individual subjects, an other in context and an other in population.

### (d) Data measurements

We manually measured four acoustic parameters from all calls using Raven interactive sound analysis (v. 1.2.1, Cornell Lab of Ornithology, Ithaca, New York) using the spectrogram window (window type: Hann; 3 dB filter bandwidth: 124 Hz; grid frequency resolution: 2.69 Hz; grid time resolution: 256 samples): duration (s), maximum frequency (Hz), maximum power (uncalibrated dB) and maximum time. Duration was the time difference between call end and onset. Maximum frequency was the frequency with maximum energy (i.e. power, dB) in a call. Maximum power was the power of the maximum frequency. Maximum time was the moment when the maximum power occurred proportional to the total duration of a call (e.g. max. time = 0.5 means it occurred half-way through the call's duration). These parameters have been found to be strong descriptors of orangutan calls and their informational content [25,28,33]. Critically, they were extractable from both consonant- and vowel-calls, enabling direct comparison between acoustic and information broadcast performance between the two call categories.

### (e) Data analyses—acoustic performance

To assess acoustic broadcast performance during transmission, linear mixed models (LLMs) (model type: III sum of squares; test model terms: Satterthwaite, using restricted maximum-likelihood) were conducted using JASP [34] (v. 0.14.1). One model was generated per acoustic parameter (×4) per call type (×2), with a total of eight models. Per model, the acoustic parameter was inserted as dependent variable (*N* = 3560 per call type). Distance (treated as ordinal: 0, 25, 50, 75, 100 m), transect (two levels), context (three levels: towards human observers, tiger-patterned predator-model, plain-white predator-model) [29] and population (three levels: Tuanan, Gunung Palung, Sampan Getek) were inserted as fixed effect variables. Individual (20 levels) and call number (*N* = 249 per call type) were inserted as random effect, since some calls were re-used for different playbacks and from the same individual. Random slopes for distance and transect were allowed to vary per individual. No explicit indication of nested variables (e.g. individual within population) was provided since this is automatically identified by the model (see [25] and electronic supplementary material).

### (f) Data analyses—information performance

To assess information broadcast performance, we conducted discriminant function analyses (DFAs) per distance [33]. All analyses were based on the four measured acoustic parameters simultaneously. Six analyses were conducted to test information content (×3; individual ID, context, population ID) for each call type (×2). LMM results indicated that ‘transect’ had a significant effect on acoustic performance over distance, hence, all (p)DFA analyses were conducted using one transect only. We conducted DFAs with leave-one-out procedure using SPSS (IBM SPSS Statistics, v. 27; electronic supplementary material) to assess information content about individual ID (same context used across individuals). To assess information content about context and population, we performed permuted DFAs (pDFAs) with cross-classification [35]: crossed pDFA for context (to control for individual variation) and nested pDFA for population (individual variation nested within population; electronic supplementary material). pDFAs were conducted in R [36] with MASS [37] and using a function provided by Mundry & Sommer [35]. Because crossed pDFAs do not tolerate null data, only three individuals with calls in all contexts were included. Figures were prepared using ggplot2 [38] and gridExtra [39]. A script example was: pdfa.res = pDFA.crossed (test.fac = ’Context’, contr.fac = ’Individual’, variables = c (Duration’, ‘Max frequency’, ‘Max time’, ‘Max power), n.to.sel = NULL, n.sel = 100, n.perm = 1000, pdfa.data = test.data).

## 3. Results

### (a) Acoustic performance over distance

**Figure 1.**
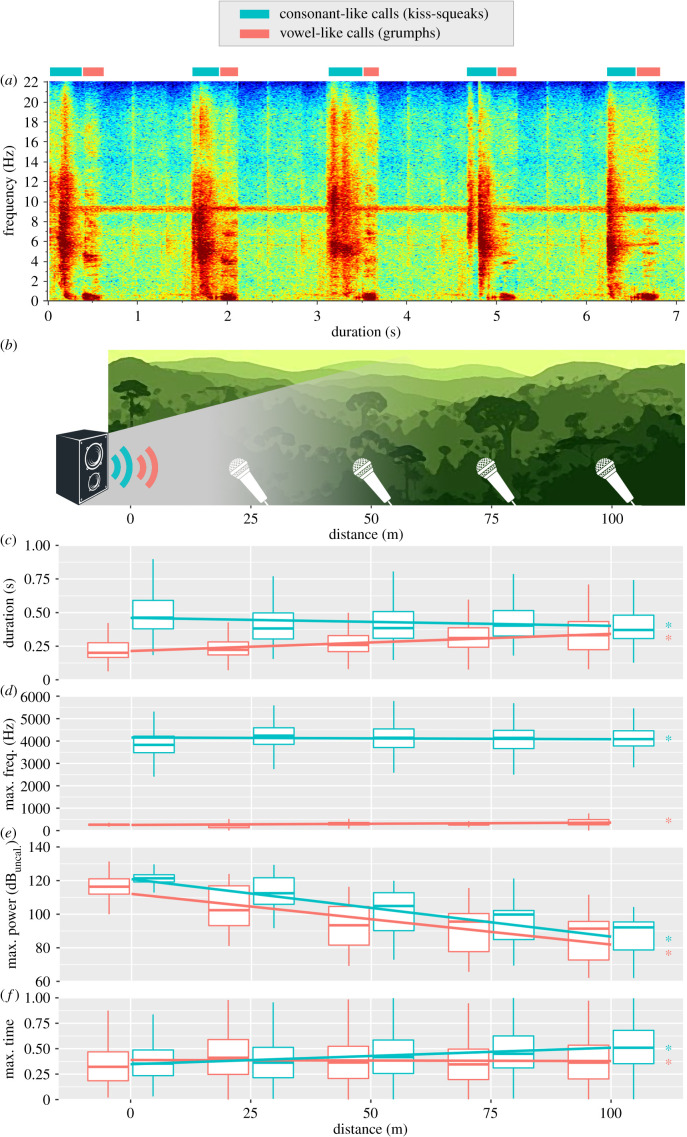
Spectrographic representation of orangutan consonant-like and vowel-like calls (*a*), graphic representation of the experimental set-up (*b*), and acoustic performance during transmission (*c*–*f*), (based on raw data). uncal., uncalibrated. Box plots represent median and 25–75% interquartile range; whiskers represent lowest/highest value within 1.5 times interquartile range below/above; outliers omitted for clarity. Linear trend lines represented across distance are for visual aid only (based on raw data). *p < 0.001 (LMM ANOVA; see table 1).

Consonant-like and vowel-like call acoustic parameters changed significantly during transmission (table 1 and figure 1, electronic supplementary material). This was expected since different parameters interact differentially with the environment (e.g. max. power declines over distance following the general inverse square law of sound attenuation). Several significant differences were found between transects (electronic supplementary material), confirming that acoustic performance was (partly) dictated by the physical structure of the transmission channel. The context had a significant effect on the acoustic performance of some parameters (electronic supplementary material). Given that both call types are known to exhibit marked contextual variation [25], this shows that the acoustic features of different contextual sub-types affect how their transmission plays out. For both consonant-like and vowel-like calls, population had a significant effect on some acoustic parameters (electronic supplementary material), suggesting that geographical accents [25] may endow calls with better transmission properties. Given that forest structure is no longer pristine across virtually all orangutan sites, it is unclear whether these gains can be attributed to adaptive selection in some populations.

**Table 1.** Acoustic performance over distance: LMM ANOVA summary.

	consonant-like calls (kiss-squeaks)			vowel-like calls (grumphs)		
	d.f.	*F*	*p*-value	d.f.	*F*	*p*-value
duration (s)	4, 16.81	14.492	<0.001	4, 20.35	51.298	<0.001
max. frequency (Hz)	4, 19.22	8.453	<0.001	4, 14.11	17.600	<0.001
max. power (dB^uncalibrated^)	4, 21.34	1825.322	<0.001	4, 23.79	1140.558	<0.001
max time	4, 14.29	28.214	<0.001	4, 19.25	9.693	<0.001

### (b) Information performance over distance

**Figure 2.**
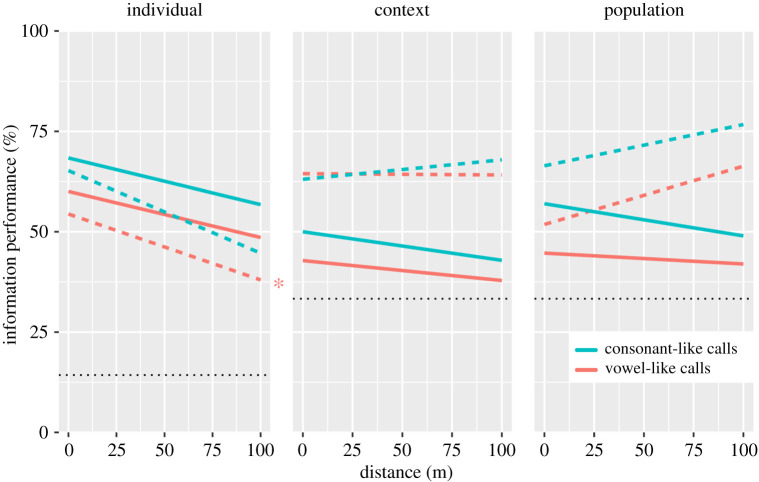
Graphical representation of information performance of orangutan consonant-like and vowel-like calls during transmission, as measured by percentage of correctly assigned cases over distance. Black dotted lines: chance level. (*a*) Continuous lines: correctly classified cases (DFA); dashed lines: correctly classified cross-validated cases (DFA leave-one-out). (*b*,*c*) Continuous lines: correctly cross-classified cases (pDFA); dashed lines: correctly classified selected cases (pDFA). *p < 0.05 (Spearman's correlation; see table 2).

Despite poor acoustic performance, informational performance of consonant- and vowel-like calls was not affected during transmission (figure 2). Both call categories allowed correct assessment of information about individual identity, context and population well above chance levels (figure 2). Information loss was only observed for individual identity when transmitted by vowel-like calls; however, this effect was only observed when computing a leave-one-out DFA procedure (a more stringent model) and information performance remained overall above chance (table 2; electronic supplementary material). Information performance was equivalent between consonant- and vowel-like calls; their trend lines remained relatively parallel over distance (figure 2). Consonant-like calls tended to exhibit higher percentage of correct assignments, suggesting heavier information load (figure 2).

**Table 2.** Information performance over distance: Spearman's correlation summary (*n* = 5). norm: correlation based on % correctly classified selected cases using DFA; L1out: correlation based on % correctly cross-classified using DFA with leave-one-out procedure; selec.: correlation based on % correctly classified selected cases using pDFA; cross: correlation based on % correctly cross-classified cases using pDFA. Italic type indicates *p* < 0.05.

	consonant-like calls (kiss-squeaks)						vowel-like calls (grumphs)					
	individual		context		population		individual		context		population	
	norm	L1out	selec.	cross	selec.	cross	norm	L1out	selec.	cross	selec.	cross
Spearman's *ρ*	−0.9	−0.8	0.6	−0.5	0.9	−0.6	−0.7	−*1*	−0.3	−0.8	0.8	−0.5
*p*	0.083	0.133	0.35	0.45	0.083	0.35	0.233	*0.017*	0.683	0.133	0.133	0.45

## 4. Discussion

We found no evidence for information loss in the only nonhuman living hominid that combines consonant-like and vowel-like calls to produce syllable-like combinations. Information content remained uncompromised until either call type became inaudible, indicating that homologous proto-linguistic units would have remained functionally discriminable as long as they could be heard. Results refute, therefore, mathematical predictions for linguistic evolution.

Orangutan consonant-like calls exhibited extreme spectral differences compared with their vowel-like counterparts (i.e. frequency centred at approx. 4000 versus 250 Hz, respectively, figure 1*a*,*d*). However, both can be information-dense [25] and their information performance was equivalent. This suggests that similar results would have been likely if other nonhuman hominid consonant- and vowel-like calls had been selected. Our analyses covered a wide frequency band wherein the actual (but now extinct) proto-linguistic units of language probably lay.

Information loss was assessed by measuring calls' biometric information content (i.e. about individual ID, context and population ID). There is no evidence that other types of informational content (e.g. culturally conventionalized arbitrary information, such as a word's meaning) transmit differently via the same acoustic signals. Some orangutan consonant-like calls exhibit arbitrary function [40] and other great ape consonant-like and vowel-like calls are transmitted culturally [7,10,11,41–46]. Thus, these calls are not unescapably limited to the transmission of biometric information, even though this was the information used for our empirical validation.

Findings offer three insights into language origin and linguistic evolution. First, proto-consonants and -vowels encoded ample information [25] and were resilient against information loss up to 100 m distance across channels adverse to signal transmission.

Second, the structural complexity of our first linguistic ancestors' habitat was an unlikely source of transmission error and information loss. Palaeo-climate change across African habitats brought about major habitat structural changes, and with them, new soundscapes. Open habitats (e.g. savannah) offer few physical obstructions to signal transmission, thus, ecological changes happening across Africa are predicted to have diminished channel noise in language's precursor system, not the opposite. Systematic assessment will be required for conclusive resolution.

Third, mathematical and computational approaches to language evolution have not, thus far, explicitly or implicitly modelled hominid behaviour. Theoretically, current models could apply to any communication system transitioning to a combinatorial state, not necessarily within the hominid family. The fact that language transpired in the human clade, but none other, implies, thus, that ‘being a hominid’ cannot be discounted from theoretical incursions that might stand a chance to enlighten us as to how linguistic evolution ensued from the repertoire of an ape-like ancestor [47]. While current models assuredly encapsulate a possible evolutionary scenario, this was not the one to have likely catalysed language. The most beneficial future theoretical models will be those that conform with, and factor in, the (consonant-vowel-based) combinatorics shared between great apes and humans.

## Data accessibility

All data needed to evaluate the conclusions in the paper are present in the paper and/or the electronic supplementary material.

## References

[1] Shannon C. 1948 A mathematical theory of communication, part I, part II. Bell Syst. Tech. J. **27**, 623-656. (10.1002/j.1538-7305.1948.tb00917.x)

[2] Szathmáry E, Smith JM. 1995 The major evolutionary transitions. Nature **374**, 227-232. (10.1038/374227a0) 7885442

[3] Lieberman P, Klatt D, Wilson W. 1969 Vocal tract limitations on the vowel repertoires of rhesus monkey and other nonhuman primates. Science **164**, 1185-1187. (10.1126/science.164.3884.1185) 4976883

[4] Boë LJ et al. 2017 Evidence of a vocalic proto-system in the baboon (*Papio papio*) suggests pre-hominin speech precursors. PLoS ONE **12**, e0169321. (10.1371/journal.pone.0169321) 28076426 PMC5226677

[5] Boë L-J et al. 2019 Which way to the dawn of speech?: reanalyzing half a century of debates and data in light of speech science. Sci. Adv. **5**, eaaw3916. (10.1126/sciadv.aaw3916) 32076631 PMC7000245

[6] Fitch TW, Boer B, Mathur N, Ghazanfar AA. 2016 Monkey vocal tracts are speech-ready. Sci. Adv. **2**, e1600723. (10.1126/sciadv.1600723) 27957536 PMC5148209

[7] Lameira AR. 2017 Bidding evidence for primate vocal learning and the cultural substrates for speech evolution. Neurosci. Biobehav. Rev. **83**, 429-439. (10.1016/j.neubiorev.2017.09.021) 28947156

[8] Lameira AR, Shumaker RW. 2019 Orangutans show active voicing through a membranophone. Scient. Rep. **9**, 12289. (10.1038/s41598-019-48760-7) PMC6707206 31444387

[9] Lameira AR, Hardus ME, Mielke A, Wich SA, Shumaker RW. 2016 Vocal fold control beyond the species-specific repertoire in an orang-utan. Scient. Rep. **6**, 30315. (10.1038/srep30315) PMC4962094 27461756

[10] Lameira AR et al. 2013 Orangutan (*Pongo* spp.) whistling and implications for the emergence of an open-ended call repertoire: a replication and extension. J. Acoust. Soc. Am. **134**, 2326. (10.1121/1.4817929) 23967963

[11] Lameira AR et al. 2015 Speech-like rhythm in a voiced and voiceless orangutan call. PLoS ONE **10**, e116136. (10.1371/journal.pone.0116136) 25569211 PMC4287529

[12] Ramsier MA, Cunningham AJ, Finneran JJ, Dominy NJ. 2012 Social drive and the evolution of primate hearing. Phil. Trans. R. Soc. B **367**, 1860-1868. (10.1098/rstb.2011.0219) 22641824 PMC3367701

[13] Quam R et al. 2015 Early hominin auditory capacities. Sci. Adv. **1**, e1500355. (10.1126/sciadv.1500355) 26601261 PMC4643776

[14] Ghazanfar AA. 2008 Language evolution: neural differences that make a difference. Nat. Neurosci. **11**, 382-384. (10.1038/nn0408-382) 18368042

[15] Schlenker P, Chemla E, Zuberbuhler K. 2016 What do monkey calls mean? Trends Cogn. Sci. **20**, 894-904. (10.1016/j.tics.2016.10.004) 27836778

[16] Hopkins WD et al. 2017 Genetic factors and orofacial motor learning selectively influence variability in central sulcus morphology in chimpanzees (*Pan troglodytes*). J. Neurosci. **37**, 5475-5483. (10.1523/JNEUROSCI.2641-16.2017) 28473646 PMC5452339

[17] Lameira AR, Call J. 2018 Time-space–displaced responses in the orangutan vocal system. Sci. Adv. **4**, eaau3401. (10.1126/sciadv.aau3401) 30443595 PMC6235548

[18] Watson SK et al. 2020 Nonadjacent dependency processing in monkeys, apes, and humans. Sci. Adv. **6**, eabb0725. (10.1126/sciadv.abb0725) 33087361 PMC7577713

[19] Townsend SW et al. 2016 Exorcising Grice's ghost: an empirical approach to studying intentional communication in animals. Biol. Rev. Camb. Phil. Soc. **92**, 1427-1433. (10.1111/brv.12289) 27480784

[20] Snowdon CT. 2009 Plasticity of communication in nonhuman primates. In Advances in the study of behavior (eds N Marc, Z Klaus, CS Nicola, JM Vincent), pp. 239-276. Cambridge, MA: Academic Press.

[21] Nowak M, Krakauer D, Dress A. 1999 An error limit for the evolution of language. Proc. R. Soc. Lond. B **266**, 2131-2136. (10.1098/rspb.1999.0898) PMC1690318 10902547

[22] Plotkin JB, Nowak MA. 2000 Language evolution and information theory. J. Theor. Biol. **205**, 147-159. (10.1006/jtbi.2000.2053) 10860707

[23] Nowak M, Krakauer D. 1999 The evolution of language. Proc. Natl Acad. Sci. USA **96**, 8028-8033. (10.1073/pnas.96.14.8028) 10393942 PMC22182

[24] Blumenthal SA et al. 2017 Aridity and hominin environments. Proc. Natl Acad. Sci. USA **220**, 201700597. (10.1073/pnas.1700597114) PMC5514716 28652366

[25] Lameira AR et al. 2017 Proto-consonants were information-dense via identical bioacoustic tags to proto-vowels. Nat. Hum. Behav. **1**, 0044. (10.1038/s41562-017-0044)

[26] Lameira AR, Maddieson I, Zuberbuhler K. 2014 Primate feedstock for the evolution of consonants. Trends Cogn. Sci. **18**, 60-62. (10.1016/j.tics.2013.10.013) 24238780

[27] Lameira AR. 2014 The forgotten role of consonant-like calls in theories of speech evolution. Behav. Brain Sci. **37**, 559-560. (10.1017/S0140525X1300407X) 25514949

[28] Hardus ME et al. 2009 A description of the orangutan's vocal and sound repertoire, with a focus on geographic variation. In Orangutans (eds S Wich, MT Setia, SS Utami, C Schaik), pp. 49-60. New York, NY: Oxford University Press.

[29] Lameira AR et al. 2013 Predator guild does not influence orangutan alarm call rates and combinations. Behav. Ecol. Sociobiol. **67**, 519-528. (10.1007/s00265-012-1471-8)

[30] Lameira AR, Call J. 2020 Understanding language evolution: beyond *Pan*-centrism. Bioessays **42**, 1900102. (10.1002/bies.201900102) 31994246

[31] Arora N et al. 2010 Effects of Pleistocene glaciations and rivers on the population structure of Bornean orangutans (*Pongo pygmaeus*). Proc. Natl Acad. Sci. USA **107**, 21 376-21 381. (10.1073/pnas.1010169107) PMC3003094 21098261

[32] Knop E, Ward PI, Wich SA. 2004 A comparison of orang-utan density in a logged and unlogged forest on Sumatra. Biol. Conserv. **120**, 183-188. (10.1016/j.biocon.2004.02.010)

[33] Lameira AR, Wich S. 2008 Orangutan long call degradation and individuality over distance: a playback approach. Int. J. Primatol. **29**, 615-625. (10.1007/s10764-008-9253-x)

[34] JASP Team. 2020 JASP (v. 0.14.1). See https://jasp-stats.org.

[35] Mundry R, Sommer C. 2007 Discriminant function analysis with nonindependent data: consequences and an alternative. Anim. Behav. **74**, 965-976. (10.1016/j.anbehav.2006.12.028)

[36] R Core Team 2013 R: a language and environment for statistical computing. Vienna, Austria: R Foundation for Statistical Computing. See http://www.R-project.org/.

[37] Venables WN, Ripley BD (eds). 2002 Survival analysis. In Modern applied statistics with S, pp. 353-385. Berlin, Germany: Springer.

[38] Wickham H. 2009 ggplot2: elegant graphics for data analysis. Berlin, Germany: Springer.

[39] Auguie B. 2012 *gridExtra: functions in Grid graphics.* *R package version 0.9*. See https://cran.r-project.org/web/packages/gridExtra/index.html.

[40] Lameira AR et al. 2013 Population-specific use of the same tool-assisted alarm call between two wild orangutan populations (*Pongopygmaeus* *wurmbii*) indicates functional arbitrariness. PLoS ONE **8**, e69749. (10.1371/journal.pone.0069749) 23861981 PMC3702587

[41] Wich SA et al. 2012 Call cultures in orang-utans? PLoS ONE **7**, e36180. (10.1371/journal.pone.0036180) 22586464 PMC3346723

[42] Taglialatela JP, Reamer L, Schapiro SJ, Hopkins WD. 2012 Social learning of a communicative signal in captive chimpanzees. Biol. Lett. **8**, 498-501. (10.1098/rsbl.2012.0113) 22438489 PMC3391466

[43] Russell JL, Joseph M, Hopkins WD, Taglialatela JP. 2013 Vocal learning of a communicative signal in captive chimpanzees, *Pan troglodytes*. Brain Lang. **127**, 520-525. (10.1016/j.bandl.2013.09.009) 24144730 PMC3982915

[44] Wich S et al. 2009 A case of spontaneous acquisition of a human sound by an orangutan. Primates **50**, 56-64. (10.1007/s10329-008-0117-y) 19052691

[45] Perlman M, Clark N. 2015 Learned vocal and breathing behavior in an enculturated gorilla. Anim. Cogn. **18**, 1165-1179. (10.1007/s10071-015-0889-6) 26139343

[46] Hayes C. 1951 The ape in our house. New York, NY: Harper.

[47] Gomez-Marin A, Ghazanfar AA. 2019 The life of behavior. Neuron **104**, 25-36. (10.1016/j.neuron.2019.09.017) 31600513 PMC6873815

